# Co-folding and RNA activation of poliovirus 3C^pro^ polyprotein precursors

**DOI:** 10.1016/j.jbc.2023.105258

**Published:** 2023-09-15

**Authors:** Grace Campagnola, Olve Peersen

**Affiliations:** Department of Biochemistry & Molecular Birology, Colorado State University, Fort Collins, Colorado, USA

**Keywords:** RNA virus, polyprotein processing, protease, protein folding, protein-RNA interactions

## Abstract

Positive-strand RNA viruses use long open reading frames to express large polyproteins that are processed into individual proteins by viral proteases. Polyprotein processing is highly regulated and yields intermediate species with different functions than the fully processed proteins, increasing the biochemical diversity of the compact viral genome while also presenting challenges in that proteins must remain stably folded in multiple contexts. We have used circular dichroism spectroscopy and single molecule microscopy to examine the solution structure and self-association of the poliovirus P3 region protein composed of membrane binding 3A, RNA priming 3B (VPg), 3C^pro^ protease, and 3D^pol^ RNA-dependent RNA polymerase proteins. Our data indicate that co-folding interactions within the 3ABC segment stabilize the conformational state of the 3C protease region, and this stabilization requires the full-length 3A and 3B proteins. Enzymatic activity assays show that 3ABC is also an active protease, and it cleaves peptide substrates at rates comparable to 3C^pro^. The cleavage of a larger polyprotein substrate is stimulated by the addition of RNA, and 3ABC^pro^ becomes 20-fold more active than 3C^pro^ in the presence of stoichiometric amounts of viral *cre* RNA. The data suggest that co-folding within the 3ABC region results in a protease that can be highly activated toward certain cleavage sites by localization to specific RNA elements within the viral replication center, providing a mechanism for regulating viral polyprotein processing.

Positive strand RNA viruses replicate their genomes in large replication center structures assembled on the surfaces of rearranged intracellular membranes. These complexes are primarily composed of viral proteins that are initially translated as large polyproteins and then processed by viral proteases into the final individual proteins and functional intermediates. Polyprotein processing plays an essential role in regulating virus replication and recruiting host factors to the viral replication centers. Poliovirus has a ≈7.5 kb genome with a single open reading frame encoding for a ∼250 kDa polyprotein that is broadly divided into P1, P2, and P3 regions. The P1 region gives rise to the four proteins that make up the viral capsid, the P2 region contains three proteins that primarily function in host membrane rearrangements and RNA packaging, and the P3 region has four proteins that are primarily involved in genome replication. The virus has two viral proteases, 2A^pro^ that only cleaves the P1/P2 region junction while 3C^pro^ carries out all other cleavages and is predominantly found in the 3CD^pro^ polyprotein form. Polyprotein processing gives rise to intermediate species that can have different functions and interaction partners than the fully processed species, expanding the biochemical functionality of the proteins encoded by the short picornaviral genome.

Within the poliovirus P3 region (Fig. 1), 3A has a ≈60-residue soluble domain, a 24-residue hydrophobic region that anchors the P3 proteins to the membrane-associated replication complexes, and a short polar C-terminal tail. This arrangement is common among picornaviral 3A proteins, although the exact lengths of the soluble regions differ and there is little global sequence conservation (1). NMR studies of the poliovirus 3A soluble region show residues 15 to 45 folding into a pair of short helices that can drive homodimerization *via* the formation of a compact four-helix bundle (2). The 3B protein, also known as VPg (viral protein, genome linked), is a short 22-residue peptide whose Tyr3 hydroxyl group becomes doubly uridylylated to generate VPg-pUpU primers for genome synthesis by the viral polymerase (3). 3C is a protease with the classic chymotrypsin double β-barrel fold and a Glu-His-Cys catalytic triad that has specificity for Gln-Gly (Q:G) sites and an additional preference for a small hydrophobic amino acid at the fourth residue N-terminal to the cleavage site (4). Last, 3D is the viral RNA-dependent RNA polymerase that is only activated upon cleavage of the 3C:3D polyprotein junction to fully structure the polymerase active site (5, 6). During virus infection, the 3CD protein is the major protease for polyprotein processing, and relatively low amounts of 3C^pro^ and active are 3D^pol^ produced. In the 3C form, the protease cleaves a wide range of host proteins to disrupt host cell functions, including vesicle trafficking SNARE proteins and nuclear pore complexes (7). 3C and 3CD also have RNA-binding characteristics that localize them to RNA elements in both the 5′ and 3′ UTRs (8, 9, 10) and to a conserved stem-loop known as the *cis*-replication element (*cre*) that templates the double uridylylation of VPg in the context of a viral replication center (11, 12).

**Figure 1:**
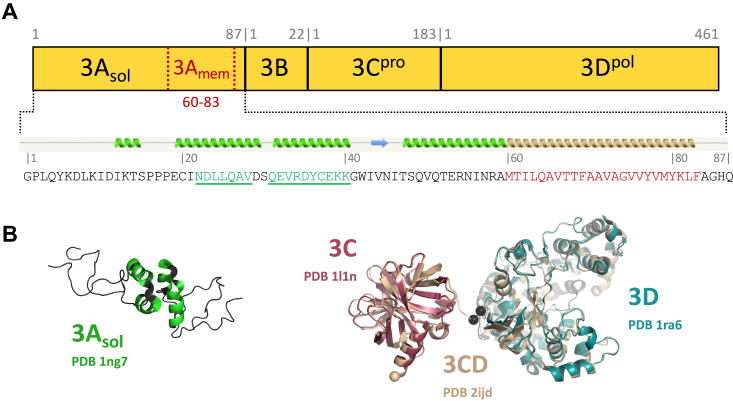
**Poliovirus P3 polyprotein region and structure.***A*, overview of the P3 region with lengths of the fully processed 3A, 3B, 3C, and 3D proteins indicated. Sequence details show the complete 3A with its consensus secondary structure prediction from the Phyre^2^ server (38). The two short soluble domain helices are highlighted in *green* and the predicted membrane binding region is in *red*. *B*, structures of different segments of the P3 proteins with PDB codes indicated. NMR studies of the 3A soluble region show residues 15 to 45 folding into a pair of short helices that drive homodimerization *via* the formation of a compact four-helix bundle. The crystal structure of 3CD is composed of independent 3C and 3D domains that are essentially identical to those of the individual structures of 3C and 3D.

The major poliovirus P3 region polyprotein processing pathway has an initial cleavage of the 3B:3C junction to generate 3AB and 3CD^pro^ proteins that are observed as stable long-lived species in infected cells. Polyprotein processing modulates host and virus protein interactions, particularly by the 3A and 3AB proteins (1). VPg uridylylation requires 3A:3B cleavage as 3AB is not a good substrate for *in vitro* uridylylation while 3B and 3BC are (13, 14, 15). Within 3A, residues 6 to 10 interact with the host factor GBF1 to inhibit protein trafficking in an interaction that requires 3A homodimerization, implicating the formation of a larger protein–protein complex during virus infection (16, 17). Residues 15 to 58 of poliovirus and kobuvirus 3A have been shown to structurally wrap around the Golgi Dynamic (GOLD) domain of the cellular ACBD3 protein, effectively anchoring this host factor to the viral replication centers (18). In these GOLD domain complexes the two short helices in 3A remain folded and homodimerize, resulting in (3A:ACBD3)_2_ tetramers that are anchored to membranes *via* the 3A hydrophobic region. Some viral proteins must be integrated into replication centers in precursor forms and then be cleaved *in situ* for proper function, for example, 3D^pol^ must enter the replication complex as the 3CD^pro^ precursor at a minimum but is more efficiently incorporated as the complete P3 protein (19). This suggests the 3A and 3B segments within P3 interact with themselves and/or the 3C, 3D, and 3CD regions to stabilize conformations important for replication center assembly and polyprotein processing.

To address the molecular mechanisms underlying these observations, we have examined the solution structure, self-association, and biochemical activities of a series of poliovirus P3 region polyprotein constructs. Our data indicate that 3AB plays a key role in stabilizing the conformation of the 3C^pro^ domain *via* an *in-cis* interaction that implicates co-folding within the 3ABC polyprotein. The resulting 3ABC is an active protease (3ABC^pro^) that has activity and selectivity comparable to 3C^pro^ on peptide substrates. However, in the presence of stoichiometric *cre* RNA the 3ABC^pro^ protease is 20-fold more efficient than 3C^pro^ at cleaving the 3B:3C junction of a folded polyprotein substrate, showing 3ABC-RNA complexes can regulate viral polyprotein processing pathways.

## Results

### Protein purification and oligomerization

The membrane binding 3A, 3AB, and 3ABC proteins were expressed from bacterial codon-optimized genes using two different pET-26-based vectors; the first has an N-terminal 11-residue ybbR tag (D**S**LEFIASKLA) that allows for CoA-linked fluorophore labeling at its Ser2 residue *via* Sfp synthase (20), and the second is an N-terminal ubiquitin (Ub) fusion that often increases protein expression and solubility. The Ub can be removed by the ubiquitin protease Ubp1 *in vivo* during bacterial expression (21) or *in vitro* with ubiquitin-specific protease-2 (USP2), and both enzymes leave an N-terminal glycine that mimics the native N-terminus resulting from viral polyprotein cleavage at Gln-Gly junctions.

The full-length P3 polyprotein (3ABCD) with an inactivating C147A mutation in the 3C^pro^ active site did not express well in bacteria and what little material could be affinity purified rapidly precipitated. In contrast, a shorter 3ABC construct expressed at high levels in CodonPlus (Agilent) cells, and about one-third of the material was recoverable with NP-40 detergent. Ub-fusions of the smaller 3A and 3AB proteins similarly expressed at high levels and for these there was complete recovery of material by detergent extraction. We also generated several 3A deletion constructs to examine protein folding, including 3A_sol_ and 3A_sol_BC that contained only the 60-residue 3A soluble region and 3A_mem_BC that contained only the 24-residue 3A membrane binding region (Fig. 1). Any proteins containing the 3A membrane binding region required detergent extraction after cell lysis, and we found 1% NP-40 was significantly more efficient than several other commonly used detergents, *e.g.*, β-octyl-glucoside, TWEEN 20, and deoxycholate. Following extraction and initial metal-affinity purification in the presence of NP-40, we examined a series of more chemically defined detergents for their abilities to solubilize 3A_mem_-containing proteins without affecting 3C^pro^ activity (Fig. S1). Based on these results, we chose 0.5 mM dodecylmaltoside (DDM) as our standard detergent for all purification steps after the initial lysis and NP-40 extraction.

**Figure 2:**
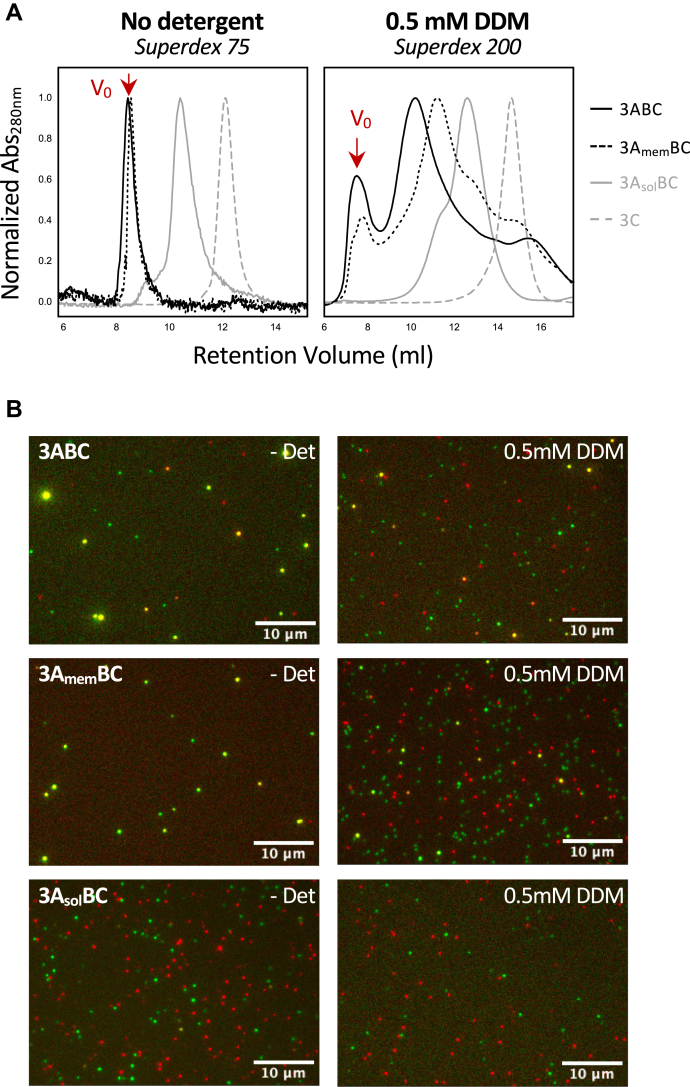
**Oligomerization states of P3 proteins.***A*, size exclusion chromatography of P3 proteins in the absence and presence of DDM detergent. In the absence of detergent proteins containing 3A_mem_ elute entirely at the void volume as large soluble aggregates, but these are disrupted by the addition of DDM to yield distinct micelle-associated species. The non-membrane-associated proteins 3A_sol_BC and 3C elute according to their monomeric molecular weights in both the presence and absence of DDM. *B*, single-molecule TIRF microscopy visualization of protein aggregation states *via* two-color labeling where dimer or large complexes appear *yellow* while monomers are either *red* or *green*. In the absence of detergent, 3ABC forms large aggregates, 3A_mem_BC forms smaller multimers, and 3A_sol_BC is mostly monomeric. Adding 0.5 mM DDM detergent solubilizes the larger aggregates into mostly dimers and monomers.

The association states of the purified proteins were assessed by size exclusion chromatography (Fig. 2*A*) and single molecule microscopy (Fig. 2*B*). In the absence of detergent, the 3ABC and 3A_mem_BC proteins eluted from a Superdex 75 size-exclusion column as large assemblies at the void volume, but notably these assemblies were soluble without any tendency to precipitate even upon being concentrated. In contrast, the non-membrane binding 3A_sol_BC and 3C proteins eluted at retention volumes consistent with their monomeric molecular weights. In the presence of 0.5 mM DDM detergent and using a bigger pore Superdex 200 column to better separate the larger protein–micelle complexes, the majority of the 3ABC and 3A_mem_BC proteins shifted out of the void volume and now eluted as smaller species with peaks at apparent molecular weights of ≈200 and ≈150 KDa, respectively. It is difficult to interpret the oligomerization state from these values because of the variable sizes and hydrodynamic shapes of micelle-associated protein complexes, but defined shoulder peaks in the chromatograms do suggest certain distinct species exist in solution (Fig. 2*A*). 3A_sol_BC eluted with a leading-edge shoulder that we attribute to a dimeric species based on the prior NMR data indicating 3A can dimerize (2) and the observation that the leading edge peak was more prominent at higher protein concentrations. Peak area analysis of multiple chromatograms from samples loaded at 10 to 30 μM concentrations showed the leading-edge peak contained 10 to 30% of the total absorbance, yielding an estimated solution dimerization K_d_ in the 20 to 60 μM range.

To directly visualize the oligomerization states of the various protein complexes we used single molecule microscopy and Sfp synthase to label the viral proteins with ATTO-565 and/or ATTO-647N fluorophores in the ybbR sequence (20). Purified fluorescently labeled proteins were diluted from ∼200 nM to ∼5 pM concentration and immediately spotted onto glass cover slips and imaged by single-molecule total internal reflectance (TIRF) microscopy to observe individual protein complexes as they irreversibly bound to the glass surface over a timecourse of several minutes (Fig. 2*B*). We labeled the samples with an equimolar mixture of two fluorophores such that dimer or greater complexes would be randomly labeled with both colors, yielding a qualitative measure of the species present in solution as they land on the cover slip; monomeric proteins show low-intensity single-molecule spots of a single color, small multimers show higher intensities of both single and dual color complexes, and larger oligomers yield large intense dual color foci.

In the absence of detergent, 70 to 80% of 3ABC and 3A_mem_BC particles were large and bright dual-color complexes (Fig. 2*B*), consistent with the chromatography elution as large oligomers at the void volume. The addition of DDM detergent clearly disrupts the large complexes (Fig. 2*B*), and ∼30% of the observed particles are now dimers or small oligomers based on counting particles and classifying them as co-localized by having overlapping centroid positions in the two color images (Fig. S1*B*). The 3C and 3BC proteins show little self-association in either the absence or presence of detergent, consistent with their SEC elution profiles as monomeric species under both conditions. 3A_sol_BC also did not show self-association in the TIRF data despite showing some dimerization in size exclusion chromatography, and we attribute this to the weak interaction that would not show dimers at the low picomolar concentrations used in the single molecule microscopy assay. Protease assays also show 3C^pro^ retains activity in DDM both below and above its 0.17 mM critical micelle concentration (CMC), indicating the protein remains properly folded (Fig. S1*C*).

### 3C conformation analysis

**Figure 3:**
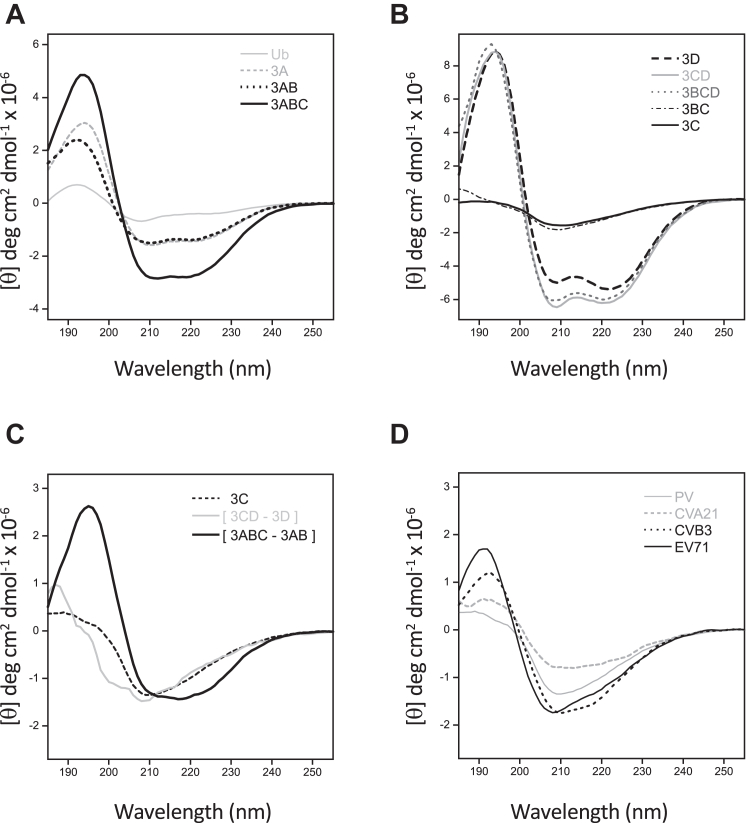
**Circular dichroism spectra of P3 region proteins.***A*, spectra of 3A, 3AB, and 3ABC, obtained from fusion proteins with an N-terminal ubiquitin whose CD signal (*solid grey line*) has already been subtracted. *B*, spectra for 3D, 3CD, 3BCD, 3BC, and 3C proteins that do not require ubiquitin for solubility. *C*, comparison of the spectrum from native 3C with difference spectra reflecting 3C in the context of 3CD and 3ABC polyproteins. A typical β-sheet type spectrum with a significant ≈195 nm peak and 215 nm minimum is only observed in the 3ABC context, while damped β_II_ type spectra are observed for 3C alone and in the 3CD and 3BC (panel *B*) contexts. *D*, a range of CD spectrum shapes are observed for 3C proteases from poliovirus, coxsackievirus A21, coxsackievirus B3, and enterovirus 71.

To examine the solution conformation of the P3 region proteins we carried out circular dichroism spectroscopy (CD) using a combination of direct measurements from the various polyprotein constructs and indirect measurements *via* difference spectrum analysis. Because we could not express and purify the full-length P3 region, the CD analysis is based on two sets of polyproteins: Figure 3*A* shows spectra for constructs growing from the N-terminal end, *i.e.*, 3A→3AB→3ABC, while Figure 3*B* shows data for constructs growing from the C-terminal end, that is, 3D→3CD→3BCD. Figure 3*B* also shows data for the internal 3BC and 3C polyprotein segments. All CD spectra were collected in 0.5 mM DDM and chloride-free perchlorate buffer to minimize background absorbance in the 190 to 210 nm range. The presence of an N-terminal ubiquitin greatly enhanced expression, purification yield, and the solubility of the membrane-binding proteins, and we therefore retained the Ub fusion on the proteins themselves, but unless otherwise noted the CD spectra shown in the figures are *after* subtracting the spectrum of the 76-residue ubiquitin. From the 3A end (Fig. 3*A*), full-length 3A showed a mixture of α-helix and random coil with a clear 195 nm maximum and minima at 208 and 222 nm. 3AB had a similar spectrum in the 200 to 240 nm region, but its 195 nm peak was reduced by ∼30%, indicating the added 3B sequence contributes mostly random coil signal. 3ABC exhibited a significant signal increase associated with adding the 183-residue 3C^pro^, yielding a typical mixed α/β structure CD spectrum with a clear maximum at 195 nm and double minima at 208 and 222 nm. From the 3D end (Fig. 3*B*), the large 461-residue polymerase also showed a typical mixed α/β structure, but interestingly the addition of 3C to make the 3CD protein only changed the signal in the 205 to 230 nm region and did not significantly alter the ≈195 nm peak. The further addition of the short 22-residue 3B to make 3BCD caused only small changes in the spectrum, again indicating 3B has a random coil conformation.

The contrast between the significant spectral changes observed when 3C was added to 3AB (Fig. 3*A*) and the small changes associated when 3C was added to 3D (Fig. 3*B*) suggests polyprotein context affects the folding and/or solution dynamics of the 3C domain. We therefore set out to compare the CD spectra of the 3C domain obtained in different protein contexts. The CD spectrum of 3C alone with native N- and C-termini was unexpectedly damped in magnitude and deviated significantly from that predicted for its 183 residues of mostly β-strand structure (Fig. 3, *B* and *C*); the prototypical β-structure maximum at 195 nm and minimum at 215 nm were both absent and the spectrum was instead unusually flat with a shallow minimum at 209 nm. This spectral shape was also seen in the [3CD-3D] difference spectrum (Fig. 3*C*), in the spectrum of 3BC (Fig. 3*B*), in spectra from 3C constructs with modified N- (*ybbr-His*_*6*_*-*3C) or C- (3C*-His*_*6*_) termini (Fig. S2*A*), and it was unaffected by the addition of DDM detergent (Fig. S2*A*). In contrast, the 3C domain spectrum obtained as the [3ABC – 3AB] difference spectrum shows a strong 195 nm maximum and a broad minimum centered on ∼215 nm (Fig. 3*C*) that is consistent with the 55% β-strand and ∼10% α-helix content observed in the 3C crystal structure (22) (PDB: 1L1N). These data show that an unusual and damped CD spectrum is characteristic of 3C as an isolated domain, and it is only in the context of 3ABC that we observe the expected β-rich spectrum for the 3C region.

### Comparison with other viral 3C proteins

CD spectra arising from β-sheets can be highly variable and dependent on subtle changes in sheet structure and twist (23), and in this context, the PV 3C^pro^ spectrum is best described as being of the β_II_ type that has a weak maximum around 190 nm and a strong minimum in the 200 nm region (24). Interestingly, such β_II_ type CD spectra have been observed for trypsin and chymotrypsin (24) that have the same overall fold as 3C^pro^, but more typical β-sheet spectra have been reported for human rhinovirus 14 and Coxsackievirus B3 3C proteins (25, 26). This prompted us to express and purify 3C from three other enteroviruses (EV71 and Coxsackieviruses A21 and B3) and compare their CD spectra obtained under identical conditions. As shown in Figure 3*D*, the four 3C proteins have distinct CD spectra, with the CVB3 and EV71 proteases showing canonical β-rich spectra with ≈195 nm maxima, 208 nm minima, and 215 nm inflection points while the PV and CVA21 proteases exhibit more β_II_ type spectra with a weak 195 nm transition. These data demonstrate that the picornaviral 3C proteins have conformational plasticity which leads them to exhibit a continuum of different β-type CD spectra while having essentially identical chymotrypsin-like overall folds.

### Deletion analysis of poliovirus 3ABC co-folding requirements

**Figure 4:**
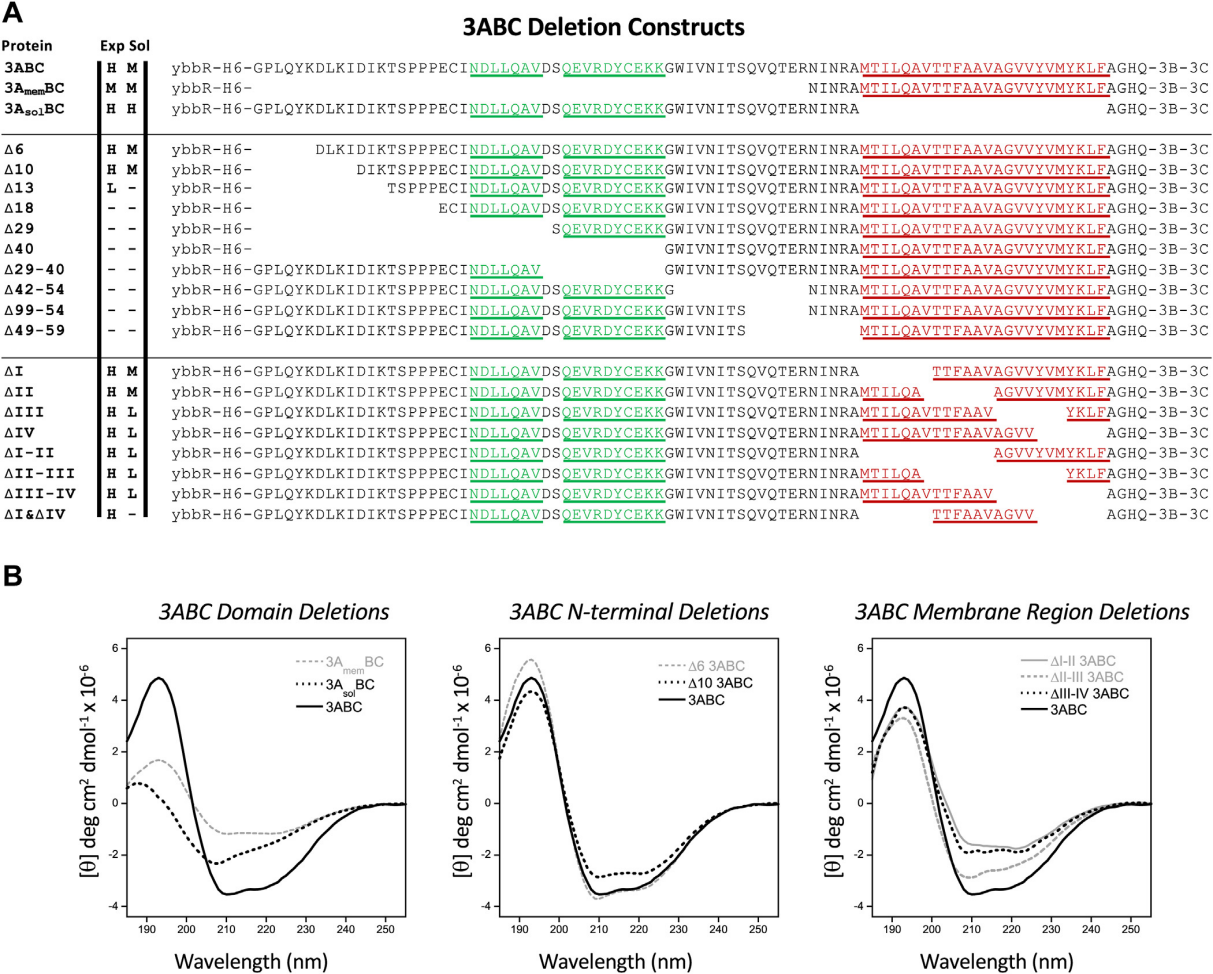
**Solubility and structure of deletions within 3A region of 3ABC.***A*, complete sequences of the 3A region from all the 3ABC proteins generated. The bacterial expression levels and solubility of each is listed on a Low/Medium/High scale with “–” indicating no protein was observed. *B*, CD analysis of deletion mutants to examine sequence elements needed for co-folding to stabilize the 3C conformation. *Left*; deletion of either the 3A_sol_ or 3A_mem_ regions will trigger the damped CD spectrum for the 3C region. *Middle*; deletion of 6 and 10 N-terminal residues do not greatly alter the structure while larger deletions could not be expressed and purified. *Right*; partial deletions within the 3A membrane binding region result primarily in loss of helix signal, with greater effects arising from deleting the first and last halves than deleting the middle of the region. Notably, the ≈195 nm peak remains strong, implying that the 3C region is unaffected by the partial deletions within 3A_mem_, unlike the complete 3A_mem_ deletion in the *left panel*.

The distinct transition of the 3C domain CD spectrum from the β_II_ type for the isolated 3C to the prototypical β type in 3ABC suggests there are folding interactions in the polyprotein context that act to stabilize the 3C conformation. To further dissect the interactions between 3A and 3C, we generated 18 3ABC deletion constructs, expressed them in *Escherichia coli*, and obtained CD spectra from the ones that could be purified in sufficient quantities (Fig. 4*A*). Deletion of either the entire 3A soluble domain (residues 1–59, yielding 3A_mem_BC) or just the membrane binding domain (residues 60–83, yielding 3A_sol_BC) resulted in a significant reduction in the 195 nm peak and a transition to the β_II_ type spectrum (Fig. 4*B*). Further analysis shows the 3A_mem_ region to be predominantly helical with a strong 197 nm maximum and 208/222 nm double minima in the [3A – 3A_sol_] difference spectrum (Fig. S2*B*). Deconvolution using DichroWeb (27) and BeStSel (28) yield ≈16 and ≈42 helical residues for 3A_sol_ and 3A, respectively, consistent with the presence of two short α-helices within 3A_sol_ (2) and the entire 3A_mem_ region being helical.

Removal of 6 and 10 N-terminal residues from 3ABC was tolerated with minor changes in the CD spectra (Fig. 4*B*), but proteins with larger deletions of 13, 19, 28, or 40 residues or internal deletion of the region between the two short helices and 3A_mem_ (residues 42–59) did not express in *E. coli*, suggesting protein folding and stability may have been affected. To assess the importance of the 3A_mem_ region we divided it into four different 7-residue deletion segments (I-IV) that each represent two turns of the predicted membrane-binding α-helix, and these were examined as individual deletions and as adjacent pair deletions (*i.e.*, 14 residues and 4-helix turns per deletion). Unlike the internal deletions in 3A_sol_, deletions within 3A_mem_ all expressed well in bacteria, although their purification recovery was lower than that of the wildtype 3ABC. The ΔI-II and ΔIII-IV deletions had the most deleterious effects on the spectra with ≈50% loss in ellipticity and a reduction in helical content (Fig. 4*B*). The central ΔII-III combination showed a more native-like spectrum, suggesting that in the context of 3ABC, a deletion of the middle portion of the 3A membrane binding region is less deleterious than deletion of its ends. But notably, none of these partial 3A_mem_ deletions triggered the transition to the β_II_-type spectrum that the full-length 3A_mem_ deletion did, indicating that the co-folding interactions do not involve the membrane binding regions.

### 3C^pro^ and 3ABC^pro^ protease activity

**Figure 5:**
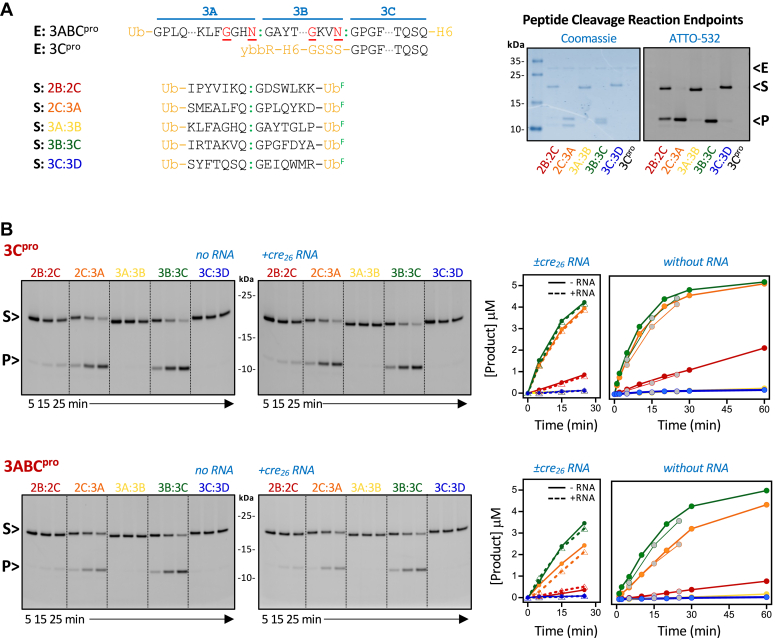
**Polyprotein junction site cleavage by 3C and 3ABC proteases.***A*, details of enzyme (E) and substrate (S) constructs, including 3ABC^pro^ mutations made to prevent self-cleavage (*red*, *underlined*) and the short N-terminal tag on 3C^pro^ to separate it from 3C product on gels. A series of substrates were made wherein 14-residue segments containing the various P2 and P3 region polyprotein cleavage sites were placed between two ubiquitin molecules, the C-terminal of which was labeled with ATTO-532 to provide a fluorescence-based cleavage assay. The gel shows reaction endpoint samples of all five junctions stained by Coomassie to visualize all species or imaged by fluorescence to visualize only the substrate and the C-terminal product. Indicators show enzyme (E), substrate (S), and product (P) bands. *B*, 3C^pro^ and 3ABC^pro^ cleavage of the five junction sites using 1 μM enzyme and 5 μM substrates. Gels and corresponding plots show that cleavage does not differ significantly in the absence (*solid lines*) and presence (*dotted lines*) of 1.5 μM *cre26* RNA. Experiments without RNA were replicated with longer reaction times, as shown by matching line colors in the plots at *right*.

To examine if the conformational differences in the 3C domain affected protease activity we assayed cleavage of both peptide substrates and polyprotein substrates using both 3C^pro^ and 3ABC^pro^ as enzymes. 3C is also an RNA binding protein and we therefore examined if adding RNA affected activity, using both a 26 nt stem-loop RNA corresponding to the tip of the PV *cre* RNA element (11, 29) and a 15 nucleotide long poly(A) RNA. Figure 5*A* shows the sequence details of the enzymes, including the mutations made at the 3ABC^pro^ polyprotein junctions to prevent self-cleavage and the five peptide substrates with viral cleavage junctions from the poliovirus polyprotein, *i.e.*, 2B:2C, 2C:3A, 3A:3B, 3B:3C, and 3C:3D sites. For these assays, we sandwiched 14-residue peptides containing seven residues on each side of the Q:G cleavage site between a pair of 76-residue ubiquitin monomers that provide distinct migration patterns for substrates and products on SDS-PAGE. In addition, the C-terminal ubiquitin was fluorescently labeled at a surface K63C mutation (R. Cohen, *personal communication*), allowing us to visualize cleavage reaction progress by both fluorescence scanning and Coomassie staining of SDS gels. Figure 5*B* shows cleavage data for all five polyprotein junctions by both enzymes in the absence and presence of *cre*_26_ RNA (5 μM substrate, 1 μM enzyme). These data show dramatic differences in cleavage efficiencies among the substrates, with a strong preference for the 2C:3A and 3B:3C junctions followed by slower cleavage of the 2B:2C junction and very slow but detectable cleavage of the 3A:3B and 3C:3D junctions. The polyprotein junction preference order is the same for 3C^pro^ and 3ABC^pro^ enzymes, indicating there is no major change in substrate recognition between the two, but the 3C^pro^ enzyme is consistently about 1.5-fold faster than 3ABC^pro^. The addition of 1.5 μM *cre*_*26*_ RNA did not significantly change the cleavage rates for either enzyme on any of the five isolated peptide junctions (Fig. 5*B*).

**Figure 6:**
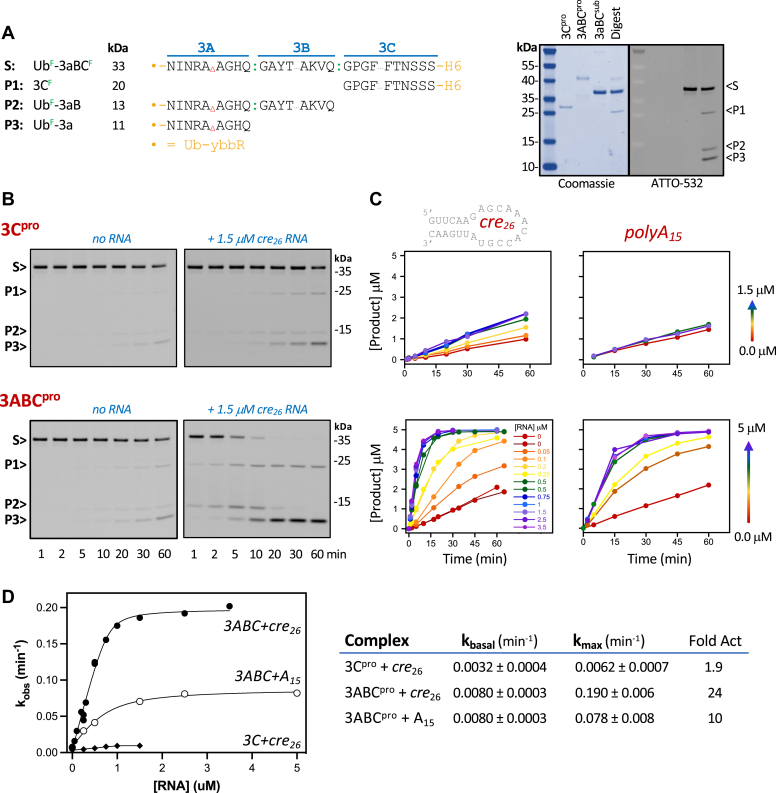
**RNA activation of polyprotein cleavage by 3C**^**pro**^**and 3ABC**^**pro**^**.***A*, sequences of the polyprotein substrate that contains both the 3A:3B and 3B:3C cleavage sites with full-length 3BC but only part of 3A (Δ marks deletion of 3A_mem_ region and the little “a” in 3aBC denotes a partial 3A protein). The substrate has two cysteine fluorophore labeling sites(^F^) to allow for visualization of the products formed during the proteolysis assay. The gel shows the two purified enzymes, the substrate, and a partially cleaved substrate stained with Coomassie and imaged by fluorescence; note Coomassie does not stain the small 3aB and 3a product bands. *B*, fluorescence images of SDS-PAGE resolved protease reaction products with 3C and 3ABC enzymes in the absence and presence of 1.5 μM *cre* RNA. Bands for substrate (S) and products 3C (P1), Ub-3aB (P2), and Ub-3a (P3) are indicated, and note the 3C labeling efficiency is ≈20% that of the Ub, making the 3C product band appear less intense. *C*, progress curves for the buildup of 3B:3C site cleavage in reactions containing increasing amounts of *cre*_*26*_ and polyA_15_ RNA (per color gradient). *D*, quadratic binding isotherm curve fits of single exponential k_obs_ values from data in (*C*) as a function of RNA concentration and fitted rate constants for basal and maximal RNA stimulated activities.

To examine activity on a folded polyprotein substrate, we made a Ub-3aBC protein that contained both 3A:3B and 3B:3C junctions with full-length 3B and 3C protein sequences but only a short piece of 3A (reflected by a lower case “a” in this “3aBC” nomenclature), and whose protease active site was inactivated by a C147A mutation (Fig. 6*A*). The 3a portion of the substrate was built on a membrane region deletion construct and contains the five residues before 3A_mem_ (NINRA) and the four residues after it (AGHQ). The N-terminal Ub was fluorescently labeled, allowing us to visualize and quantify the short Ub-3a and Ub-3aB products that are too small to observe by Coomassie staining, and the 3C portion was fluorescently labeled at a surface T120C mutation to also visualize the C-terminal cleavage product. Note that the labeling efficiency of this site is low and as a result the 3C product bands are fainter than the N-terminal Ub fragments. Figure 6*B* shows the cleavage time courses obtained in the absence and presence of increasing concentrations of *cre*_26_ and *polyA*_15_ RNA. In the absence of RNA, we observe significantly slower cleavage of the 3B:3C junction than in the peptide substrates, showing that polyprotein context can modulate polyprotein processing rates, and 3ABC^pro^ is now the faster enzyme at approximately twice as fast as 3C^pro^. Intriguingly, the addition of *cre* RNA strongly stimulated activity on these polyprotein substrates and this stimulation was much stronger for 3ABC^pro^ than 3C^pro^. With 3ABC^pro^ and *cre* RNA, the 3B:3C junction is cleaved the fastest (t_1/2_ ≈5 min) to generate 3C and the 3aB intermediate that is then more slowly (t_1/2_ ≈20 min) processed into the final 3a and 3B species. The cleavage efficiencies at both junctions are thus dramatically increased by the addition of RNA.

To further characterize the RNA stimulation we carried out replicate assays with multiple RNA concentrations using both the 26-nt tip of the viral *cre* RNA and a generic 15-nt poly(A) RNA (Fig. 6*C*). The resulting data show clear concentration dependencies for both RNAs. We extracted rate constants (k_obs_) for each RNA concentration by fitting the data to a single exponential curve assuming an endpoint of 5 μM product, plotted these k_obs_ values as a function of RNA concentration and fit those data to an RNA activation curve using the quadradic form of the binding equation because the 1 μM enzyme and the titrated RNA are at similar concentrations. The resulting RNA activation curves show that 3ABC^pro^ cleavage of the 3B:3C junction is activated 10-fold by *polyA*_15_ RNA and 24-fold by *cre* RNA. In contrast, 3C^pro^ is not significantly stimulated by *polyA*_15_ RNA and is only 2-fold activated by *cre* RNA (Fig. 6*D*). The stimulation is saturable for both RNAs and 0.82 ± 0.05 μM *cre* RNA is needed to activate 1 μM added enzyme, suggesting that a stochiometric 1:1 protease–RNA complex is involved. We also validated the RNA activation effect using a non-fluorescent, non-ubiquitinated 3aBC substrate to verify that the ubiquitin and fluorophore modifications did not alter protease activity (Fig. S3).

## Discussion

The multifunctional nature of positive strand RNA virus polyproteins places constraints on both biochemical activities and structures whose folding must be stable in the context of a larger polyprotein, smaller cleavage products, and in some cases complexes with host factors. In this work we examined the self-association, secondary structure, and protease activity of the poliovirus 3ABCD polyprotein, *i.e.*, the P3 region, using a combination of size exclusion chromatography, single-molecule microscopy, circular dichroism spectroscopy, and protease assays. This led to the discovery of co-folding within the complete 3ABC region that internally stabilizes the conformational state of the 3C domain and results in an active 3ABC^pro^ precursor whose protease activity is greatly stimulated by the poliovirus *cre* RNA element.

The circular dichroism spectroscopy data shows co-folding within the 3ABC protein whereby the 3AB segment stabilizes the conformation of the 3C domain. The spectrum of the poliovirus 3C alone is an unusual β_II_ type, yet the comparison of 3C protein spectra from CVA21, CVB3, and EV71 show a range of conformations extending toward canonical β-sheet type spectra (Fig. 3*D*). Canonical spectra have previously been observed for human rhinovirus 14 and Coxsackievirus B3 3C^pro^ proteins (25, 26), but interestingly β_II_ type CD spectra have been observed for trypsin and chymotrypsin (24). The 3C^pro^ proteases have a chymotrypsin-like fold and catalytic triad mechanism that uses a cysteine instead of serine in the active site, and they similarly select for the amino acid at the C-terminal side of the cleaved peptide bond *via* a substrate binding pocket. There is a long-established precedent for allosteric control of chymotrypsin family proteins *via* a conformational selection mechanism in which the enzymes exist in an equilibrium between having accessible and inaccessible active sites (30, 31). These proteases exhibit a wide range of catalytic efficiencies that can in large part be attributed to the balance of this conformational equilibrium, and mutations far from the active site can allosterically enhance or diminish protease activity by altering the equilibrium.

These observations raise the question of to what extent the picornaviral 3C^pro^ proteases use this mechanism to regulate polyprotein processing during viral infections. NMR data show the poliovirus 3C^pro^ structure to be conformationally dynamic on a millisecond timescale and the binding of both ssRNA and short substrate peptides alter the distribution of structural states (32). This NMR study also showed that a 10-mer ssRNA corresponding to the 5′ side of the *cre* stem led to a slight ≈1.4-fold increase in PV 3C^pro^ activity on a short peptide substrate. Our data build on this by showing that a longer duplex stem-loop corresponding to the very tip of the viral *cre* RNA can more strongly stimulate protease activity on native polyprotein substrates and the effect is significantly greater for 3ABC^pro^ than 3C^pro^. These findings suggest that both polyprotein processing and RNA binding can act as allosteric regulators of 3C^pro^ activity during virus replication. The crystal structure of 3CD^pro^ shows two independently folded domains whose structures are essentially identical to those of the two isolated proteins (33) (Fig. 1*B*), which is consistent with our CD spectra, and both NMR (34) and molecular dynamics (35) studies indicate fairly minor differences in 3C domain protein dynamics between 3C^pro^ and 3CD^pro^ contexts. Based on this, we expect the co-folding interactions we observed within the 3ABC segment also take place within the complete 3ABCD polyprotein, *i.e.*, P3 would initially fold as a 3ABC domain and a 3D domain.

In this context, we propose that the 3ABC segment within the P3 polyprotein supplies an RNA-activated protease whose activity is effectively localized to sites within the replication center where specific RNA elements are present. The combined observations that the stimulation is twenty times greater for 3ABC^pro^ than 3C^pro^, saturates at ≈1 μM RNA that matches the enzyme concentration, and occurs on the 3aBC substrate but not the Ub-peptide substrates together imply the activation is specific to the enzyme-substrate-RNA complex. The major pathway for poliovirus P3 polyprotein processing has an initial cleavage of the 3B:3C junction and this must be an *in trans* cleavage by a simple geometric argument that the newly created 3C^pro^ N-terminus is at the end of a well-structured α-helix and ≈23 Å away from its own active site. The cleavage separates 3AB from 3CD^pro^, removing the 3ABC co-folding interaction and reducing 3C^pro^ activity *via* loss of RNA stimulation. Most of the active protease made during infection is found in the 3CD^pro^ form and localized to the replication centers, but a limited amount of 3C^pro^ is released into the cytoplasm where it cleaves a plethora of host proteins (7). Having a highly active protease run rampant inside the cell would clearly be deleterious, as exemplified by digestive proteases that are translated as inactive precursors and excreted from cells prior to activation. Our data suggest the poliovirus 3C^pro^ may be similarly autoregulated, but in the opposite direction; it is initially very active in an RNA-stimulated 3ABC form when bound to viral RNA elements in the replication center, but then has much lower activity once fully processed to the 3C^pro^ that is released into the cytoplasm. Last, co-folding within 3ABC may also act to sequester the 3A protein and limit its ability to interact with host factor proteins prior to the 3B:3C processing event; both poliovirus and kobuvirus 3A proteins have been shown to recruit the host factor ACBD3 to the membrane replication centers by wrapping around its GOLD domain in an extended conformation (18), and limiting this interaction *via* 3ABC co-folding could regulated virus replication *via* modulating host factor recruitment.

In summary, we have used circular dichroism spectroscopy and protease assays to demonstrate co-folding within the poliovirus 3ABC polyprotein region that stabilizes the structure of the 3C^pro^ region and produces a protease that is strongly activated by the viral *cre* RNA element. The detailed mechanistic understanding and specific RNA requirements of this activation will require further biochemical and structural studies, but conceptually our findings implicate RNA elements as possible site-specific regulators of polyprotein processing in the context of membrane anchored viral replication centers.

## Experimental procedures

### Protein expression and purification

All proteins used in this study were expressed from a T7 promoter using a pET-26-based plasmid in *E. coli* BL21-CodonPlus cells (Agilent Technologies, Inc) grown at 37 °C to an OD_600_ of 0.6 and then induced with 0.5 mM IPTG and grown at room temperature for an additional 18 h. Harvested cells were lysed at 18,000 lb/in^2^ in an M-110L microfluidizer (Microfluidics/IDEX Corp.) in a buffer containing 50 mM Tris pH 8.0, 300 mM NaCl, 10 mM imidazole, and 20% glycerol. The lysate from soluble proteins, that is, those that did not contain any part of the 3A membrane binding region, were centrifuged for 30 min at 17,000 rpm in a Beckman JA-21 rotor, and the clarified lysate was loaded onto a 5-ml HisTrap FF column (Cytiva) followed by step elution with 50 mM Tris pH 8.0, 300 mM NaCl, and 350 mM imidazole. Fractions containing the desired proteins were pooled and diluted 4-fold into a buffer containing 25 mM Tris pH 8.5, 50 mM NaCl, 20% glycerol to reduce the ionic strength for ion exchange chromatography on a HiTrap Q column (Cytiva) to remove contaminating nucleic acid. Proteins that did not bind the Q-column were recovered from the flow through and proteins that bound were gradient eluted with 25 mM Tris pH 8.5, 1 M NaCl, and 20% glycerol. Fractions were pooled and concentrated to ∼500 ul prior to SEC purification on Superdex 75 or 200 Increase 10/300 Gl columns equilibrated with 200 mM NaCl, 50 mM Tris pH 7.5, and 20% glycerol. Purified proteins were stored at −80 °C.

For proteins containing any part of the 3A membrane binding region (3A_mem_), the cells were lysed in the same imidazole buffer stated above supplemented with 1% NP-40 and incubated for 2 h at 4 °C with gentle rocking prior to centrifugation. HisTrap and Q column fractions were collected into tubes pre-loaded with sufficient 10 mM DDM (Anatrace) for a 0.5 mM final concentration while the gel filtration columns were run in 200 mM NaCl, 50 mM Tris pH 7.5, 20% glycerol, and 0.5 mM DDM.

### Protein fluorophore labeling at the ybbR site

20 mM Coenzyme A (New England Biolabs) was first labeled overnight at 30 °C with equimolar amounts of ATTO-dye maleimides (ATTO-TEC GmbH) in 400 mM Tris, pH 7.5 and the reactions were quenched by diluting 10-fold into the same buffer containing 5 mM DTT. The 565- and 647N-CoA were purified on a 1 ml HiTrap Q column (Cytiva) equilibrated in 50 mM Tris pH 8.5 with washing until the excess fluorophore was removed and elution by a NaCl gradient. The final 565-CoA or 647N-CoA concentration was determined by fluorophore absorption on a Cary Bio50 spectrophotometer. Sfp synthase expression plasmid (generously provided by Dr Jeff Knight, University of Colorado – Denver) was transformed into *E. coli* BL21-CodonPlus cells that were grown at 37 °C to an OD_600_ of 0.6 and then induced with 0.5 mM IPTG to express protein at room temperature for 6 h. The harvested cells were lysed and HisTrap affinity purified as described above. Column fractions containing the protein were pooled and diluted 4-fold into a buffer containing 25 mM Tris pH 8.5, 50 mM NaCl, and 20% glycerol to reduce the ionic strength for ion exchange chromatography on a HiTrap Q column (Cytiva). Q fractions were pooled, and the Sfp synthase was concentrated to 170 μM and stored in aliquots at −80 °C.

Proteins containing the N-terminal ybbR tag (D**S**LEFIASKLA) were fluorescently labeled using Sfp synthase (4′ -phosphopantetheinyl transferase) (36) in reactions containing 50 mM Tris 7.5, 10 mM MgCl_2_, 40 mM NaCl, 20 μM ATTO-565 and/or ATTO-647N labeled CoA, 1 μM Sfp synthase and ∼10 μM substrate protein. Reactions were incubated at room temperature for 2 h then placed at 4 °C overnight and excess CoA-fluorophore was removed by size exclusion chromatography as stated above. Fluorophore labeling efficiency was typically 75 to 85% for the soluble protein constructs and 20 to 40% for the membrane-binding proteins.

### TIRF association and detergent selection

Fluorescently labeled proteins in the indicated detergent concentrations were added to 50 μl CoverWell imaging chambers (Grace Bio-Labs) adhered to piranha protocol cleaned coverslips. Proteins were visualized by objective-type total internal reflection fluorescence microscopy on a Nikon Ti-Eclipse microscope using a 1.49 NA 100× Plan Apo TIRF lens and an iXon3 DU897 EM-CCD camera (Andor). Protein association was determined as a percent of co-localized particles using the ComDet plug-in (https://github.com/ekatrukha/ComDet) for ImageJ as implemented in Fiji (37).

### Circular dichroism experiments

Proteins were buffer exchanged using Superdex 75 or 200 Increase 10/300 Gl columns equilibrated in a low absorbance buffer containing 150 mM sodium perchlorate, 0.5 mM DDM, and 10 mM Tris pH 7.0 whose pH was adjusted with H_2_SO_4_. The eluted proteins were immediately used for CD experiments and concentrations were determined based on 280 nm absorbance and extinction coefficients calculated by the ProtParam tool at Expasy (http://web.expasy.org/protparam/). CD, absorbance, and PMT voltage data were collected at room temperature using a MOS-500 spectrometer (Bio-Logic) scanning 185 to 265 nm with a 2 nm slit width at an acquisition period of 2 s per 1 nm step. The absorbance and PMT voltage values were kept below 1 AU and 500 V, respectively, allowing for protein concentrations of 1 to 7 μM depending on sample composition. All buffer and protein scans were collected in PTFE stoppered 1 mm UV quartz cuvettes (FireflySci Inc). Raw ellipticity values were buffer subtracted and averaged over 3 to 12 scans depending on CD signal and time-dependent spectral changes, then converted to MRE or micromolar ellipticity values. The DichroWeb (http://dichroweb.cryst.bbk.ac.uk) (27) and BeStSel (http://bestsel.elte.hu) (28) web servers were used to estimate secondary structure content from the CD data.

### Protease assays

The gene for an active Ub-3ABC^pro^ with mutated 3A:3B and 3B:3C junctions to prevent self-cleavage (Fig. 5*A*) was ordered from Twist Bioscience and cloned into a pET26 expression vector by In-Fusion Cloning (Takara). The 3C^pro^, 3ABC^pro^, and substrates were expressed and purified as described above. Protease reactions included 96% unlabeled and 4% fluorescently labeled substrate to follow reaction progress and multiple species by gel scanning. The Ub3aBC substrate was labeled at a unique surface cysteine (T120C) introduced into the 3C region, and the Ub-peptide-Ub substrates were labeled at a K63C mutation in the C-terminal ubiquitin. Purified unlabeled substrates were incubated with ATTO-532 maleimide at a 2:1 fluorophore to protein ratio (100 uM:50 uM) at room temperature for 2 h, re-purified by gel filtration using a Superdex 75 Increase 10/300 Gl column (Cytiva) to remove the excess fluorophore, concentrated to 50 to 100 μM, and stored in aliquots at −80 °C.

Protease reactions contained 1 μM enzyme, 5 μM substrate and various concentrations of the 26-nucleotide long *cre* RNA or 15-nucleotide long polyadenosine synthesized by Integrated DNA Technologies. Protease reactions were quenched at indicated times with 2× LDS gel-loading buffer (Invitrogen) supplemented with 14 mM β-mercaptoethanol. Quenched reactions were resolved on surePAGE 10 to 20% gradient gels (GeneScript) and imaged with a Typhoon RGB scanner (Cytiva) to detect fluorescent substrates and products and then stained with Coomassie Brilliant Blue R-250 and imaged with the 700 nm channel of a Li-Cor Odyssey CLx scanner. Band intensities were quantified using Li-Cor ImageStudio software and plotted as the fraction of cleavage products over time. Reaction timecourse data were curve fit to a single exponential function in Kaleidagraph (Synergy Software) to obtain observed reaction rates (k_obs_) at each RNA concentration. These values were then curve fit in Prism (GraphPad Software) to a quadratic form RNA-binding dependent activation model, with automatic outlier rejection (no data were rejected) and fitting a single common protease concentration across all datasets to determine the activated rate constants.

## Data availability

All data are contained within the manuscript.

## Supporting information

This article contains supporting information.

## Conflict of interest

The authors declare that they have no conflicts of interest with the contents of this article.
