# Supplementary material for: Co-folding and RNA activation of poliovirus 3Cpro polyprotein precursors
Source: J Biol Chem. 2023 Sep 15;299(11):105258. doi: 10.1016/j.jbc.2023.105258 (PMC10590986; doi:10.1016/j.jbc.2023.105258)
Supplement: Supporting Figures S1–S3 [file mmc1.pdf]

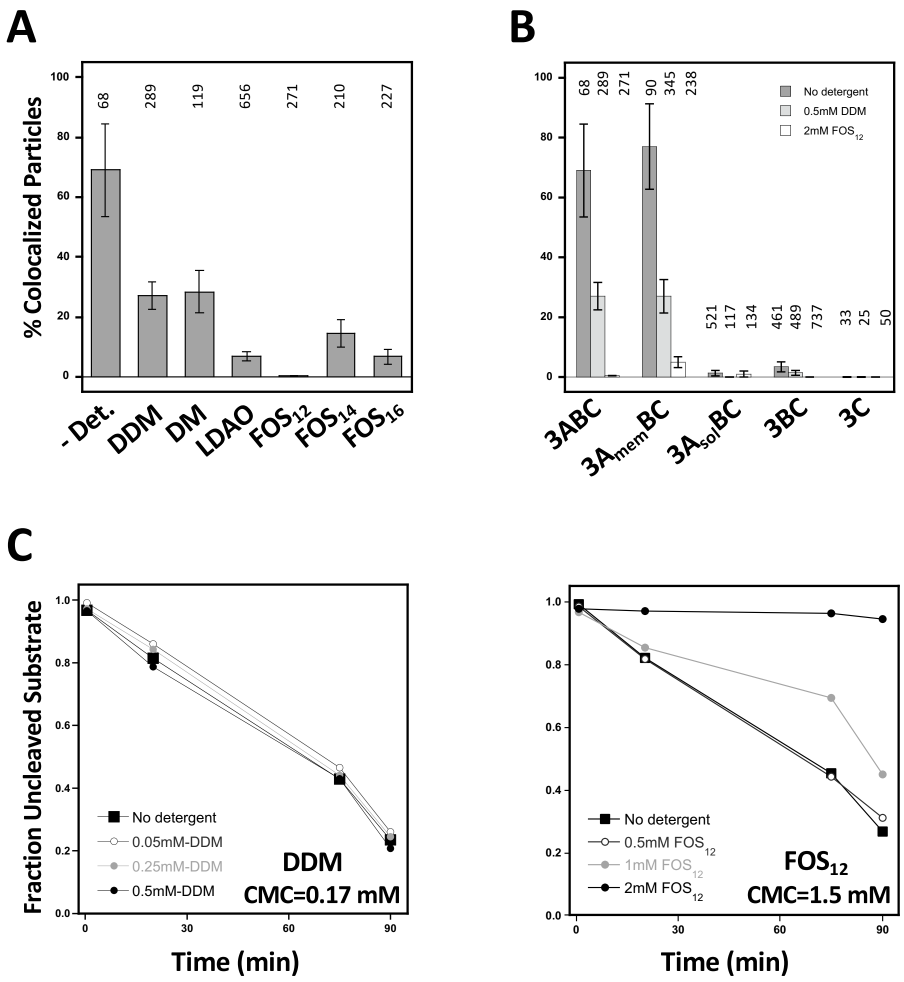

Figure S1: Detergent effects on oligomerization and protease activity.

A, Solubilization of 3ABC particles by several detergents, shown as the fraction of 3ABC molecules found in two-color particles that are indicative of dimer or larger assemblies (see Fig. 2B). DDM and DM detergents readily disrupt large particles to monomers and dimers while FOS_n detergents result in predominantly monomers. The total number of particles analyzed are indicated above each bar on the graph.

B, Effects of micellar concentrations of DDM and FOS_{12} detergents on a series of P3 protein constructs. The membrane binding 3ABC and $3A_{mem}BC$ proteins show a mixture of monomers and dimers with DDM, but only monomers with FOS_{12}. The remaining soluble protein constructs are monomeric in both the absence and presence of detergent. The total number of particles analyzed are indicated above each bar on the graph.

C, Protease data showing that DDM has no effect on $3C^{pro}$ activity, but FOS_{12} completely inhibits the enzyme at concentrations above its critical micelle concentration (CMC).

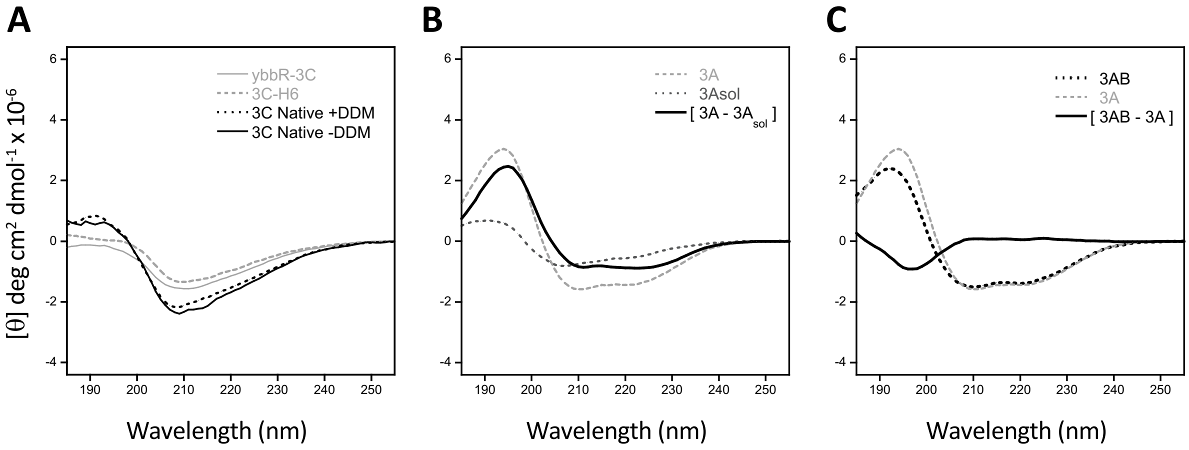

Figure S2: Circular dichroism spectra of 3C, 3A, and 3AB proteins.

A, CD spectra showing nearly identical traces for 3C proteins with native and modified N- and C-termini and in the absence and presence of 0.5 mM DDM detergent.

B, The 3A membrane binding region is predominantly α–helical based on the difference spectrum obtained by subtracting the $3A_{sol}$ region signal from that of full length 3A.

C, 3B has a mostly disordered random coil conformation based on the spectrum obtained by subtracting the 3A spectrum from the 3AB spectrum.

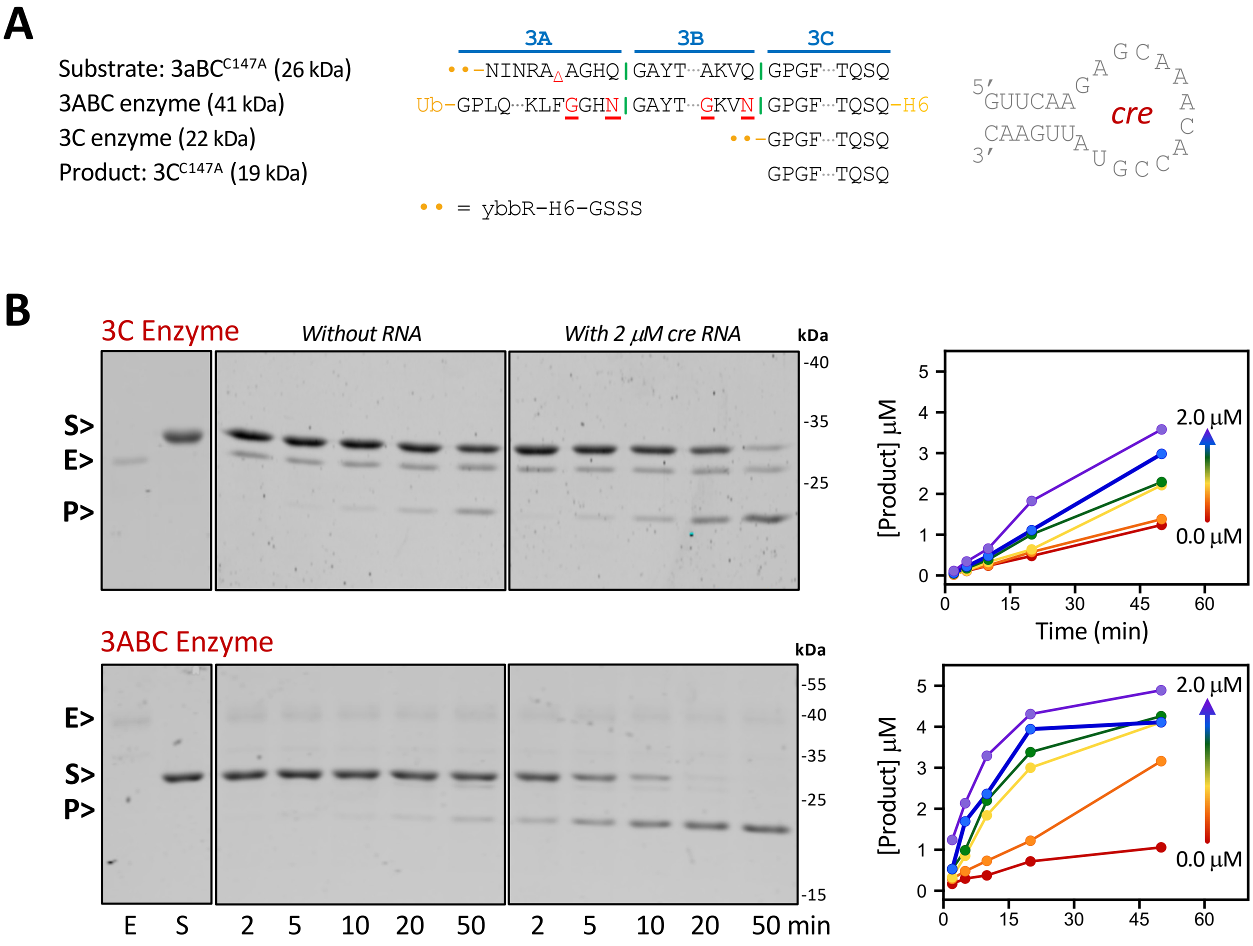

Figure S3: Non-fluorescent Coomassie stained assay for 3C and 3ABC protease activity.

A, Sequences of the polyprotein mimic substrate that contains both 3A:3B and 3B:3C cleavage sites with full length 3BC but only part of 3A (Δ marks deletion of $3A_{mem}$ region), the 3ABC enzyme in which both junctions have been mutated at red positions to prevent self-cleavage, and the 3C enzyme with a short N-terminal tag to separate it from the 3C product on gels.

B, Coomassie stained SDS-PAGE of protease reaction products with 3C and 3ABC enzymes in the absence and presence of 2 μM *cre* RNA. Bands for 1 μM enzyme (E), 5 μM substrate (S), and 3C product (P) are indicated. The membrane region of 3ABC causes the enzyme to run as a fuzzy band on the gel; its concentration in the reaction is based on UV absorbance quantitation. Plots at right show progress curves for the buildup on 3C product in reactions with increasing amounts of *cre* RNA (0, 0.1, 0.2, 0.5, 1, 2 μM).
